# Pericardial effusion and cardiac tamponade requiring pericardial window in an otherwise healthy 30-year-old patient with COVID-19: a case report

**DOI:** 10.1186/s13256-020-02467-w

**Published:** 2020-09-09

**Authors:** Christina Walker, Vincent Peyko, Charles Farrell, Jeanine Awad-Spirtos, Matthew Adamo, John Scrocco

**Affiliations:** Mercy Health St. Elizabeth Boardman Hospital, 8401 Market Street, Boardman, OH 44512 USA

**Keywords:** COVID-19, Pericardial effusion, Acute pericarditis, Cardiac tamponade, Case report

## Abstract

**Background:**

This case report demonstrates pericardial effusion, acute pericarditis, and cardiac tamponade in an otherwise healthy woman who had a positive test result for coronavirus disease 2019. Few case reports have been documented on patients with this presentation, and it is important to share novel presentations of the disease as they are discovered.

**Case presentation:**

A Caucasian patient with coronavirus disease 2019 returned to the emergency department of our hospital 2 days after her initial visit with worsening chest pain and shortness of breath. Imaging revealed new pericardial effusion since the previous visit. The patient became hypotensive, was taken for pericardial window for cardiac tamponade with a drain placed, and was treated for acute pericarditis.

**Conclusion:**

Much is still unknown about the implications of coronavirus disease 2019. With the novel coronavirus disease 2019 pandemic, research is still in process, and we are slowly learning about new signs and symptoms of the disease. This case report documents a lesser-known presentation of a patient with coronavirus disease 2019 and will help to further understanding of a rare presentation.

## Introduction

Acute pericarditis, pericardial effusion, and cardiac tamponade should be considered in patients presenting with chest pain who have suspected or proven coronavirus disease 2019 (COVID-19). Clinicians should broaden their differential diagnoses when evaluating these patients and use ultrasound early to help guide the diagnosis. Early diagnosis and treatment will likely prevent complications associated with acute pericarditis.

As more information is gathered in relation to COVID-19, the medical community will have a better understanding of the numerous clinical manifestations of the disease. For now, we must take into account various presentations, common and uncommon, related through case reports such as the present one and many others.

The goal of this report is to review and improve understanding of an uncommon complication associated with COVID-19. Additionally, one must maintain a wide differential diagnosis at all times, particularly when there is a tendency to focus on lung pathology while considering complications of COVID-19.

## Case presentation

A 30-year-old Caucasian woman with no past medical history presented to our hospital with a 3-day history of fever (maximum body temperature [TMax] 101.0 °F [38.3 °C]), dry cough, and exertional chest pain. She denied any shortness of breath, nausea, vomiting, diarrhea, dysuria, and rash. The patient works in healthcare and has had many possible exposures to severe acute respiratory syndrome coronavirus-2 (SARS-CoV-2), the novel coronavirus. She denied recent travel or immobilization. She had no risk factors or history of cardiac disease or thromboembolic disease. Her family history revealed that her mother had had a heart attack at an unknown age.

The patient’s vital signs included a heart rate of 116 beats/minute, blood pressure of 107/74 mmHg, respiratory rate of 16 breaths/minute, body temperature of 96.8 °F (36.0 °C), and oxygen saturation of 97% on room air. She was tachycardic, but she otherwise had a normal physical examination result.

**Fig. 1:**
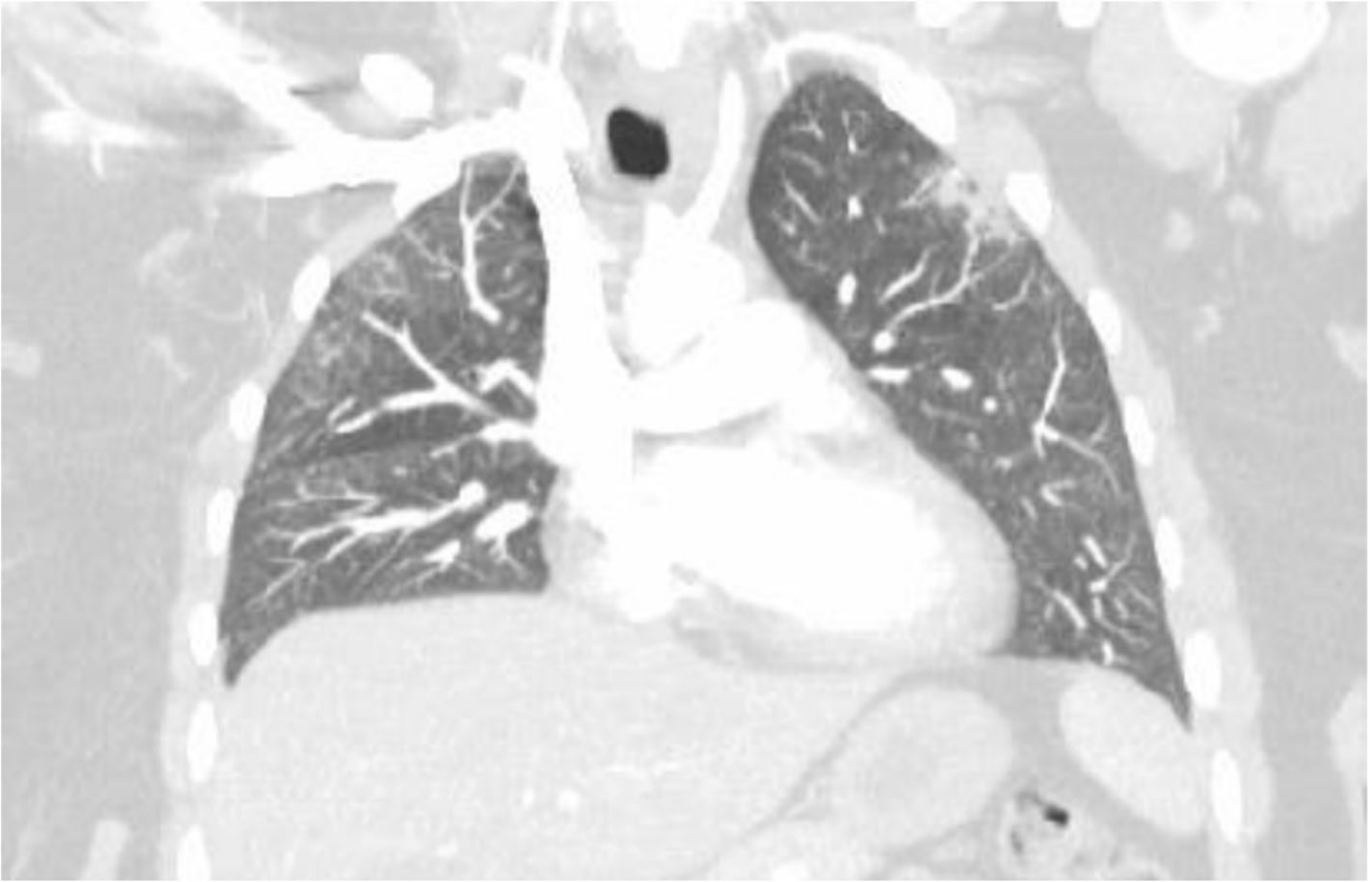
Initial CTA suggesting pneumonia and ground-glass opacities

Her electrocardiogram (EKG) demonstrated sinus tachycardia. Basic chemistry test and cardiac enzyme test results were obtained. The patient’s D-dimer was elevated at 264 ng/ml, so computed tomography angiography (CTA) was performed to evaluate for pulmonary embolism (PE). The results demonstrated no evidence of PE; however, there was evidence of interstitial pneumonia with subpleural interstitial densities and ground-glass opacities (Fig. 1).

The patient appeared well upon reevaluation. Her tachycardia had improved with intravenous fluids, and she was stable for discharge. She was written prescriptions for cefdinir and azithromycin for presumed community-acquired pneumonia treatment as well as ipratropium-albuterol.

The patient was a healthcare worker, so COVID-19 testing (Abbott RealTime SARS-CoV-2 assay; Abbott Molecular, Abbott Park, IL, USA) was performed. She was discharged to home with isolation instructions and strict return precautions. Her COVID-19 test result was positive.

Two days later, the patient returned with worsening chest pain and shortness of breath. She had been compliant with discharge medications from the previous visit. Her fever had resolved. The result of her home pulse oximetry had remained above 97%. She had developed increased tachycardia with heart rates above 130 beats/minute.

The patient’s physical examination revealed that she was well-appearing. She was tachycardic with dry mucous membranes. The remainder of the examination was unchanged. She exhibited no conversational dyspnea.

Her initial vital signs included a heart rate ranging from 116 to 134 beats/minute, blood pressure of 95/69 mmHg, respiratory rate of 18 breaths/minute, body temperature of 97.8 °F (36.6 °C), and oxygen saturation of 97% on room air. She was administered 1 L of intravenous fluids. Repeat laboratory tests and CTA of the chest were performed.

Significant laboratory values included brain natriuretic peptide of 7890 pg/ml. The patient’s EKG again showed sinus tachycardia without any obvious ST elevation or depression, no electrical alternans, and normal voltage. A respiratory polymerase chain reaction viral panel result was negative for all pathogens tested.

**Fig. 2:**
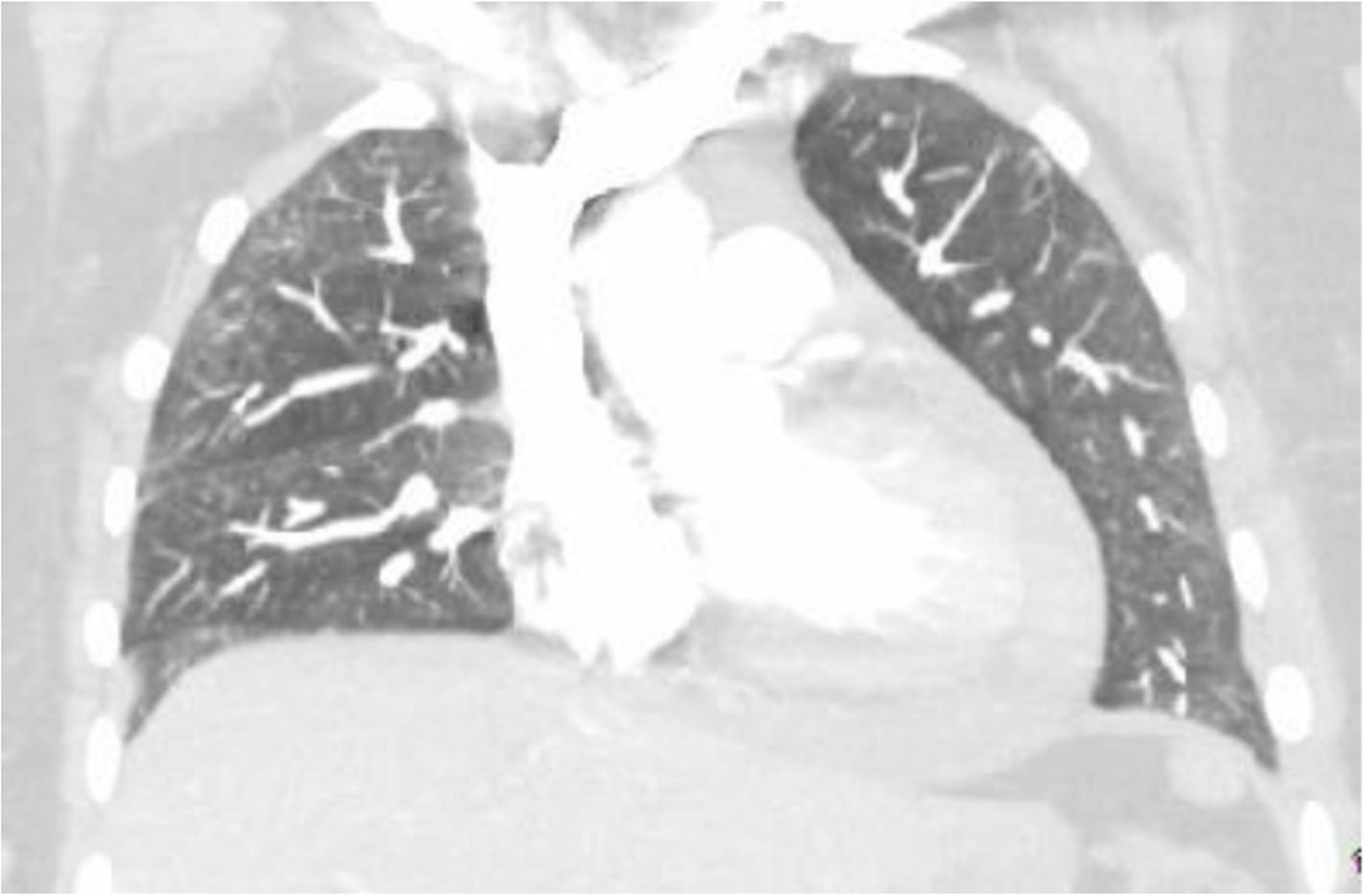
Repat CTA showing pericardial effusion

CTA showed improvement of infiltrates with no evidence of PE. It also showed the presence of a pericardial effusion that was not seen on imaging from the previous visit (Fig. 2).

**Fig. 3:**
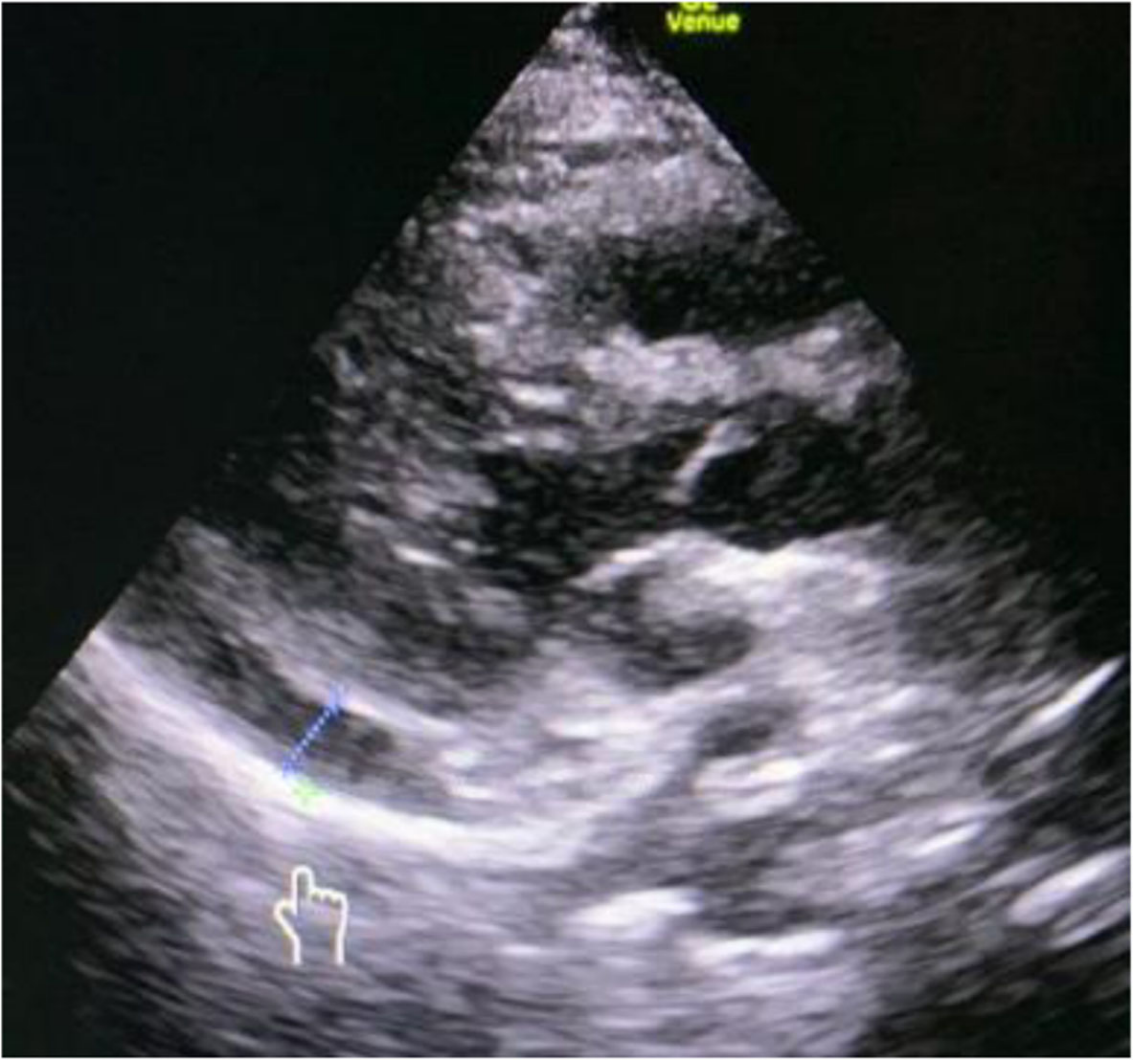
US showing pericardial effusion (*pointer*)

**Fig. 4:**
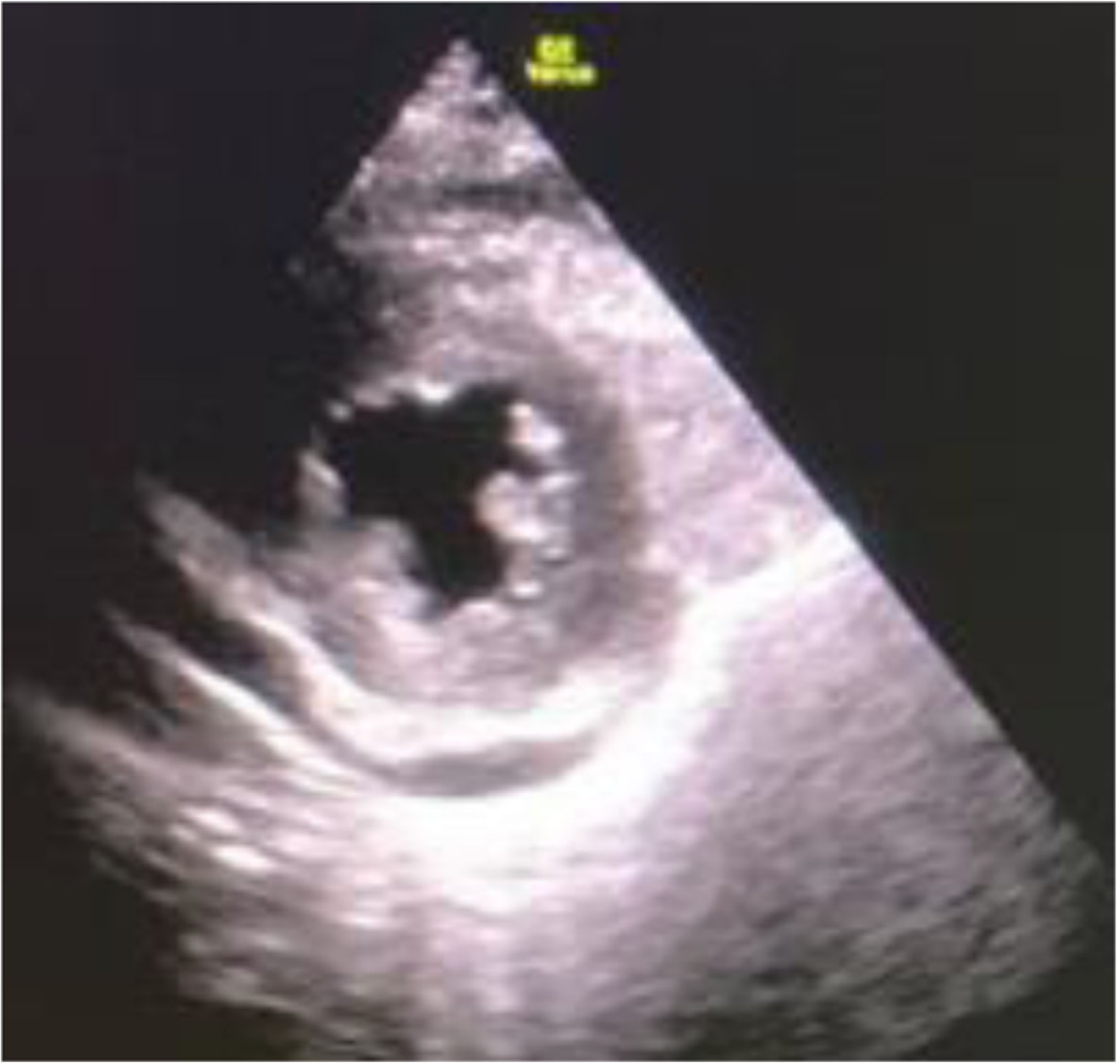
US showing pericardial effusion

In addition, an echocardiogram at that time showed a moderate-sized pericardial effusion of approximately 12 mm (Figs. 3 and 4).

Soon after this time, the patient became hypotensive with a blood pressure of 77/58 mmHg. She was administered an additional 500 ml of normal saline.

A central line was placed in the right femoral vein, and phenylephrine was initiated, which led to slight improvement of her hypotension. However, she remained persistently tachycardic with a maximum heart rate of 134 beats/minute. She developed tachypnea with a respiratory rate of 44 breaths/minute, so she was placed on 6 L of nasal cannula oxygen after arrival in the intensive care unit. She was started on hydroxychloroquine as well.

Overnight, the patient remained hypotensive and tachycardic. She continued to be tachypneic with increased work of breathing, and the following morning, she was taken to the operating room for an emergent subxiphoid pericardial window for cardiac tamponade. She had over 150 ml of strawlike output. A mediastinal drain was placed.

On postoperative day (POD) 1, the patient’s blood pressure remained stable on phenylephrine, and her tachycardia improved with a heart rate between 103 and 119 beats/minute. She was maintaining an oxygen saturation above 92% on a 3-L nasal cannula. Her pericardial drain output was 300 ml. Colchicine was initiated for acute pericarditis.

On POD 2, the patient was febrile with a TMax of 100.6 °F (38.1 °C). Her blood pressure remained stable on phenylephrine, and oral midodrine was initiated. Her tachycardia improved with a heart rate ranging from 100 to 108 beats/minute. The patient was placed on a 4-L nasal cannula, and the pericardial drain was removed.

On POD 3, the patient was afebrile. Vasopressors were discontinued, and her tachycardia improved. She remained on a 4-L nasal cannula.

On POD 4, the patient continued to experience mild chest pain and a mild cough, but she had improvement of her breathing. Her blood pressure and heart rate remained stable. She was transferred to a monitored telemetry floor with a 3-L nasal cannula.

The patient’s symptoms continued to improve during her hospital course, and she was discharged to home on POD 7 with aspirin, colchicine, and pantoprazole for treatment of viral pericarditis. She was discharged with instructions to undergo a repeat echocardiogram 2–4 weeks after a negative COVID-19 test result. The results of blood and sputum samples from both her initial emergency department visit and hospital admission were negative.

## Discussion and conclusions

This report describes a case of our patient with viral pericarditis causing a pericardial effusion resulting in cardiac tamponade secondary to COVID-19 infection. Our patient initially presented with mild symptoms and stable vital signs. She returned 2 days later with worsening tachycardia and hypotension, and she had developed a pericardial effusion. This case demonstrates that COVID-19 can affect multiple organ systems beyond respiratory complications. COVID-19, even though it enters the lung via angiotensin-converting enzyme 2, can also affect the heart and kidneys [1]. Common COVID-19 presentations are still being elucidated, which highlights the importance of all presentations of the novel infection.

A review of current evidence discusses the first case of cardiac tamponade arising from COVID-19 in a previously healthy 47-year-old woman presenting with chest pain and shortness of breath with an echocardiogram demonstrating pericardial effusion; the patient was hypotensive despite fluid repletion and eventually underwent pericardiocentesis with improvement in hemodynamic status [2]. A recent case report documented a case of acute myopericarditis with systolic dysfunction 1 week after a patient developed a fever and cough arising from COVID-19 [3]. Additionally, a recent meta-analysis reported that approximately 4.55% of chest computed tomographic scans obtained in patients with suspected or confirmed COVID-19 have shown evidence of pericardial effusion [4]. We suspect that more cases of viral pericarditis, pericardial effusion, and pericardial tamponade associated with COVID-19 exist that have gone unreported.

A systematic review of the PubMed, Embase, and WHO databases of publications discussed 919 patients with COVID-19 who developed pericardial effusion. The study described it as an uncommon finding associated with COVID-19 [5]. This conclusion was echoed by a Chinese study of 90 patients with pericardial effusion associated with COVID-19 [6]. However, we believe that pericardial effusion may be a useful clinical feature to help distinguish severe from mild disease. A review of 83 patients supports this, showing that 4 (16.0%) of 25 critical patients demonstrated pericardial effusion versus 0 (0%) of 58 patients with mild disease [7].

Acute pericarditis is diagnosed by at least two of the following four features: chest pain, a pericardial rub, saddle-shaped ST elevation and/or PR depression (sinus tachycardia with PR shortening and any depression), and nontrivial new or worsening pericardial effusion [8]. Our patient exhibited two of four features, including chest pain and pericardial effusion.

Approximately 90% of acute pericarditis cases are idiopathic or viral. Viral cultures and antibody titers are often not useful clinically [9]. Pericarditis has been described in cases of other coronaviruses, including SARS-CoV and Middle East respiratory syndrome CoV, making the diagnosis of acute pericarditis in our patient with COVID-19 reasonable [10, 11]. Thus, we felt it appropriate to continue treatment for acute pericarditis due to our patient’s COVID-19 infection.

Four days after discharge, our patient was contacted for follow-up. She was still requiring oxygen and was short of breath with exertion, but felt much better each day. She had follow-up appointments with her primary care physician and cardiothoracic surgeon scheduled for the following week.

In addition to a focus on other symptoms, prevention is important. The basic reproductive number (R_0_) is higher than previously thought [1]. This means that COVID-19 is significant and demands focused nonpharmacologic prevention strategies such as wearing masks, social distancing, quarantining, isolation, and diligent hand hygiene [12].

## Data Availability

All data generated or analyzed during this study are included in this published article and its supplementary information files.

## Abbreviations

COVID-19: Coronavirus disease 2019
CTA: Computed tomography angiography
EKG: Electrocardiogram
PE: Pulmonary embolism
POD: Postoperative day
SARS-CoV-2: Severe acute respiratory syndrome coronavirus-2
TMax: Maximum body temperature
